# A Systematic Review of the Value of a Bladder Scan in Cauda Equina Syndrome Diagnosis

**DOI:** 10.7759/cureus.14441

**Published:** 2021-04-12

**Authors:** Awf A Alshahwani, Joseph Boktor, Amr Elbahi, Purnajyoti Banerjee

**Affiliations:** 1 Trauma and Orthopaedics, Leicester University Hospital, Leicester, GBR; 2 Trauma and Orthopaedics, Cardiff University Hospital, Cardiff, GBR; 3 Trauma and Orthopaedics, Kettering General Hospital, Kettering, GBR

**Keywords:** cauda equina, bladder scan, pvr, screen, adjunct

## Abstract

Cauda equina syndrome (CES) is one of the emergency conditions that can lead to devastating permanent functional disabilities, if misdiagnosed. Multiple studies have questioned the reliability of clinical assessment in diagnosing CES, whether some of the features should be considered to be potential red flags. Bladder dysfunction can reflect CE compromise. The post-void residual (PVR) volume bladder scan is useful in CES diagnosis, but to date there has been no single systematic review supporting its use. Furthermore, there is no clear cut-off point to consider PVR statistically significant. The aim of the study is to perform a systematic review of the current evidence behind the use of the PVR bladder scan as a diagnostic tool for CES diagnosis. This was a comprehensive search using Medline, PubMed and Embase. All articles included post-void bladder scans with the mentioned clear cut-off volume as a diagnostic parameter. A total of five study articles from 1955 fit with our inclusion and exclusion criteria. The total number of patients who had a bladder scan was 531. CES was confirmed in 85 cases. Bladder scan diagnosed 70 cases and excluded 327. The best results for both sensitivity and specificity in correlation with the sample of the study were for PVR more than 200 ml. Measuring the post-void urine volume using a bladder scan is an essential tool in the diagnosis of CES. There is a significant correlation between the PVR volume more than 200 ml and higher sensitivity and specificity.

## Introduction and background

Cauda equina syndrome (CES) is an emergency spinal pathology that can lead to devastating permanent functional disabilities. It is rare with an estimated incidence of 1 in 2000 [1]. Missing timely diagnosis of cauda equina compression can end up in irreversible bowel, bladder and sexual dysfunction. Missed CES represents the most common diagnoses associated with successful litigation and paying off huge sum of compensation to the victim thereby causing substantial financial strain in every health system in the world. In the United States, nearly half of the claims have been paid, and settlement costs millions of dollars per person [2,3].

There is a debate in the literature questioning the accuracy of clinical features in the diagnosis of CES including saddle area anesthesia, perineal loss of sensation, sphincter dysfunction, bilateral sciatica, and motor and sensory function disturbances [4]. Magnetic resonance imaging (MRI) scan is essential to rule out cauda equina as no single or collective features will definitely confirm or exclude diagnosis [5]. The British Association of Spine Surgeons (BASS) emphasized that an urgent MRI scan should be performed for suspected CES, and decompressive surgery should be undertaken at the earliest opportunity in confirmed cases [6]. Early surgery is indicated to avoid the permanent functional disability and financial implications associated with a missed diagnosis or inadequate management. Therefore, early diagnosis with an MRI scan is an essential tool to achieve this [7].

As MRI scanning may not be available in peripheral hospitals with no specialist spinal surgical services, an alternative measure is to identify the patient who needs a transfer to the specialist spinal “hub” hospital where MRI scans can be undertaken especially after working hours. These hospitals usually have around-the-clock cover for spinal emergencies and might reduce the risk of further delay by undertaking an MRI scan and then surgery if necessary without the need for further consultations or transfer to another center [6]. Furthermore, exposing a patient to an unnecessary imaging study during a situation like coronavirus disease 2019 (COVID-19) pandemic and using the same machine for COVID-positive patient can result, theoretically at least, to an increased spread of the disease, in addition to the burden on the resources [8]. As bladder function can be assessed on a frequent basis through urination, post-void bladder volume represents a surrogate marker of the cauda equina function and the bladder musculature contractions as well [9]. Bladder emptying, which can be accurately assessed by a bladder scan, would be a good criterion [10].

In this study, we have undertaken a systematic review, assessing the efficacy of a bladder scan used as a bedside clinical tool in possible diagnosis of cauda equina compression. Furthermore, we have delineated an accurate cut-off point to highlight the possibility of CES and prioritize the patient for a subsequent MRI scan.

Our research question is, “Is bladder scan a suitable tool for screening cauda equina syndrome complementary to clinical examination?” and “What would be the expected significant post-void residual (PVR) volume to proceed to the MRI scan?”

## Review

**Figure 1:**
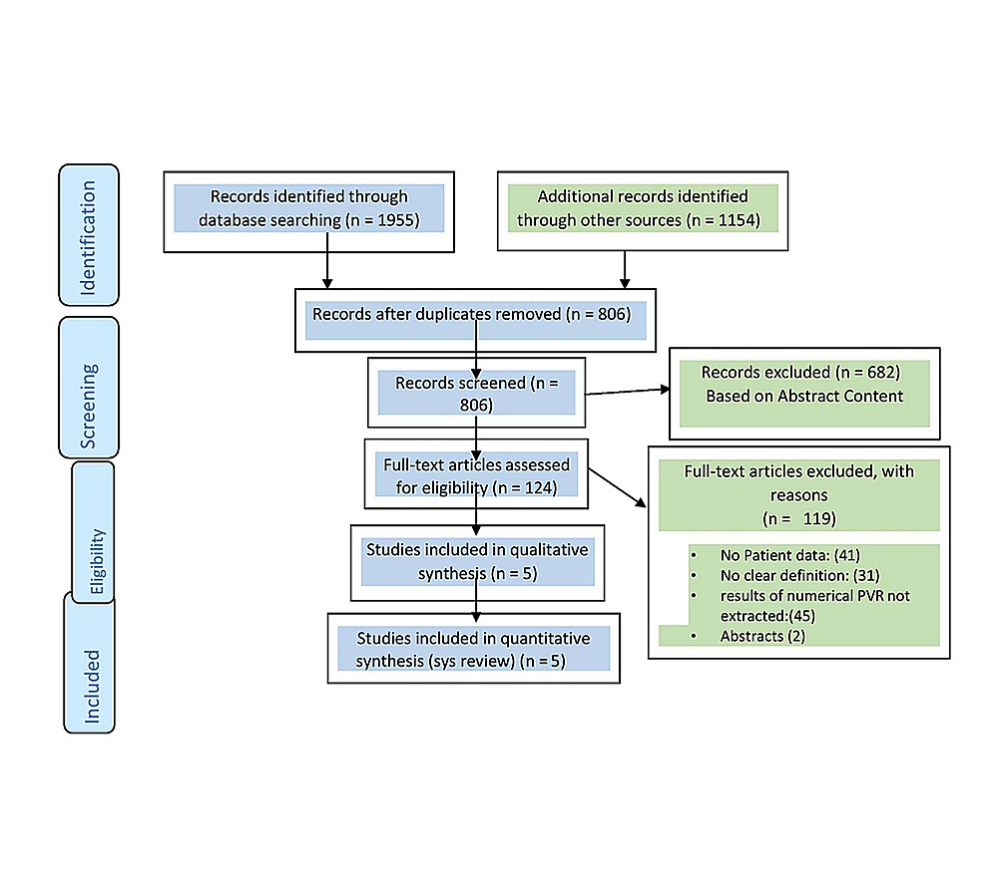
PRISMA Flow Diagram PRISMA, Preferred Reporting Items for Systematic Reviews and Meta-analyses; PVR, post-void residual

This systematic review was performed according to the Preferred Reporting Items for Systematic Reviews and Meta-analyses (PRISMA) guidelines as shown in Figure 1. 

Starting on December 2019, till September 2020, a comprehensive search was done by two spinal surgeons with the help of an experienced librarian. We looked at the databases including Embase, PubMed and Medline. We included original peer-reviewed studies that included numeric values of PVR apparent in bladder scans as a diagnostic tool and MRI scans done to confirm the diagnosis of CES. We excluded (1) abstracts, case reports and studies mentioning high post-void residual volumes but no actual numeric values reported and (2) studies that did not detail urine retention and volume but only mentioned bladder dysfunction. Filtration of studies was done by reading the article title, abstract and for some studies, by reading the full articles. The process was repeated by two authors and unrelated studies were removed after revising.

The first electronic search detected a total of 1955 studies published from 2008 till July 2020. No studies were found before 2008. Removal of duplicates was done and 806 studies remained and were further screened for eligibility based on title and abstract; 120 articles were found eligible. After reading full text, only seven studies met the inclusion criteria. Yet two of the seven were abstracts and were not fully published articles. There was another randomized controlled trial that was stopped due to ethical approval withdrawal [11].

A total of five studies were included in this review. Table 1 shows the key features of these studies. Four of the five studies were retrospective and one study was prospective. In the reviewed papers, the total number of patients who had a bladder scan was 531. CES was confirmed in 85 cases. Bladder scan diagnosed 70 cases and excluded 327. We looked at the sensitivity and specificity of each study and found the best results for both sensitivity and specificity in correlation with the sample of the study would be at PVR >200 ml as shown in Table 2.

**Table 1:** Key features of the five studies PVR, post-void residual; CES, cauda equina syndrome

Title	Author & Year	Study Type	Total No. of Cases	Cases With Positive MRI for CES	Outcome
Predictive value of clinical characteristics in patients with suspected cauda equina syndrome [12]	Domen et al. (2009)	Retrospective	58	8	A PVR volume more than 500 ml with or without other clinical features of CES is an important predictor for CES
Does rectal examination have any value in the clinical diagnosis of cauda equina syndrome? [5]	Gooding et al. (2013)	Retrospective	57	13	No single clinical feature is adequate as standalone to discriminate with statistical significance to proceed to the MRI outcome
The clinical features and outcome of scan-negative and scan-positive cases in suspected cauda equina syndrome: a retrospective study of 276 patients [13]	Hoeritzauer et al. (2018)	Retrospective	276 but only 65 had a scan	15	No PVR records for two-thirds of the patients
Bladder scans and postvoid residual volume measurement improve diagnostic accuracy of cauda equina syndrome [9]	Venkatesan et al. (2019)	Prospective	92	17	The presence of red flags with a PVR volume more than 200 ml obligates the necessity of an MRI scan
A prospective study of the role of bladder scanning and post-void residual volume measurement in improving diagnostic accuracy of cauda equina syndrome [14]	Katzouraki et al. (2020)	Prospective	260	32	Use of the PVR volume ≥200 ml was considerably more accurate in predicting CES. It is a useful adjunct to conventional clinical assessment and allows risk stratification in managing suspected CES

**Table 2:** Data analysis from the studies, with sensitivity and specificity PVR, post-void residual; TP, true positive; FN, false negative; FP, false positive; TN, true negative

Author	PVR	Patients	TP	FN	FP	TN	Sensitivity	Specificity
Hoeritzauer et al. [13]	>100	65	10	5	24	26	67%	52%
Venkatesan et al. [9]	>200	92	16	1	19	56	94%	72%
Domen et al. [12]	>500	58	6	2	0	50	100%	93%
Gooding et al. [5]	>500	57	6	7	0	44	46.1%	100%
Katzouraki et al. [14]	>200	260	32	2	75	151	94.1%	66.8%

CES was first described in the mid-50s by Jennett after correlating symptoms of back pain, sphincter dysfunction with an intraoperatively confirmed disc prolapse [15]. The urgency of decompression surgery in CES was emphasized by Ahn et al. who noticed in his meta-analysis better prognosis if decompression was done within 48 hours, and even better prognosis was detected by Kohles et al. when surgery done earlier within 24 hours [7,16].

Early decompression should be preceded by prompt workup with a confirmed diagnosis by an MRI scan as soon as possible, and since this includes detecting potential patients even outside working hours and on weekends, there must be a rationale to perform MRI scans in potential cases with all the economic and logistic implications especially in peripheral hospitals where no MRI service is available after hours and over weekends. In 2019, Dionne et al. concluded that known clinical red flag symptoms are not sensitive enough to detect CES [6,17].

Mechanical compression of S2, S3 and S4 nerve roots can be picked up early as they can cause weakness of the detrusor muscle in the bladder wall that controls voluntary bladder function and affects its emptying capability. This can be picked up early with a bladder scan and measuring the PVR volume [10]. PVR volume is utilized as a part of the screening pathway of management of back pain with bowel/bladder weakness to identify the need to involve the spine service [18,19].

Domen et al., in 2009, showed that a PVR volume >500 ml had a sensitivity of 100% and a specificity of 93%. The study involved 58 patients where all were inspected for red flags including PVR more than 500 ml; using this high value as a cut-off point carries the risk of missing impending CES that might progress with time and might lead to irreversible CES with retention with no expected recovery of the urinary function [12].

Gooding et al., four years later, used the same cut-off point when they included 57 patients with suspected CES; 13 of them (23%) were found to have CES on MRI scan. Surprisingly, they showed much less impressive conclusion with the sensitivity of PVR at 38% and the specificity 76% [5].

Hoeritzauer et al., in 2018, included 276 patients of whom only 65, a third of the patients, had recorded PVR, as the study focused on differentiating MRI scan positive and negative CES. A cut-off point of PVR >100 ml was used. The sensitivity and specificity were 67% and 52%, respectively [13].

In 2019, Venkatesan et al., in a prospective study hypothesized >200 ml PVR as the most reliable cut-off point. The study included 92 patients, which is the largest number of patients involved amongst all studies. The sensitivity was 94% and specificity 72%. The probability of CES was 43% (P<0.000003). This is an adequate evidence for the relationship between CES and PVR, with a reasonable cut-off point for PVR [9].

More recently, from the same institute in 2020, Katzouraki et al. followed 260 patients and found a similar predictive value of 200 ml with CE compression on MRI, and where candidates underwent subsequent surgery (P<0.0001, Fisher’s exact test). The PVR volume >200 ml had a sensitivity of 94% in predicting CES and specificity 66.8%, with 29.9% positive predictive value and 98.7% negative predictive value [14].

There are a number of limitations to this study. A limited numbers of articles fit with the scope of the systematic review. All studies are retrospective except for two, and there are no randomized controlled trials and meta-analysis studies.

## Conclusions

Post-void urine volume using a bladder scan is essential in the diagnosis of CES. The most suitable cut-off point with statistically significant sensitivity and specificity correlation with the study samples would be PVR >200 ml. There is a need for a large-sample size prospective trial with a multi-center input to clearly delineate the true role of bladder scan as a screening tool for CES. It is unclear how accurate a bladder scan is to dictate the need for an urgent MRI scan in early cauda equina compression (incomplete CES); however, it definitely represents a valuable adjunctive tool. Furthermore, a meta-analysis might help in determining the optimal cut-off PVR to guide when we should proceed to an MRI scan.
